# Stapled versus handsewn closure of enterotomy for intracorporeal overlap anastomosis in laparoscopic colectomy: in vitro study

**DOI:** 10.1007/s10151-025-03190-8

**Published:** 2025-07-22

**Authors:** Tetsuo Ishizaki, Junichi Mazaki, Kenta Kasahara, Ryutaro Udo, Tomoya Tago, Yuichi Nagakawa

**Affiliations:** Department of Gastrointestinal and Pediatric Surgery, Tokyo Medical University, 6-7-1 Nishi-Shinjuku, Shinjuku-ku, Tokyo, 160-0023 Japan

**Keywords:** Closure of enterotomy, Stapled closure, Handsewn closure, Anastomotic area, Intracorporeal anastomosis, Laparoscopic colectomy

## Abstract

**Background:**

In laparoscopic colectomy, overlap anastomosis (OA) is the most standard method of intracorporeal anastomosis. To avoid narrowing the anastomotic area, the closure of the enterotomy is often performed with handsewn running sutures of the monofilament. The purpose of this study was to compare two porcine in vitro colon models of stapled versus handsewn closure of enterotomy in intracorporeal OA.

**Methods:**

In total, 40 porcine in vitro colon OA models (20 cases in the stapled closure, SC group, in which the enterotomy was closed with a stapler, and 20 cases in the handsewn closure with monofilament, HC group) were created, and anastomotic area with maximum intensity projection-computed tomography, anastomotic time, and leakage pressure were measured.

**Results:**

In the anastomotic area, there was no significant difference between in the SC group and HC group (474.0 ± 105.0 mm^2^ versus 502.6 ± 155.6 mm^2^, *p* = 0.552). The anastomotic time was significantly shorter in the SC group than in the HC group (185.9 ± 38.3 s versus 292.4 ± 67.8 s, *p* < 0.001). The leakage pressure was significantly higher in the SC group than in the HC group (30.1 ± 3.8 mmHg versus 21.6 ± 5.3 mmHg, *p* < 0.001).

**Conclusions:**

The findings of this study using porcine in vitro colon model showed that, in OA, the anastomotic area was similar, anastomotic time was significantly shorter, and leakage pressure was significantly higher in SC compared with HC. The results suggest that SC may be superior to HC when performing intracorporeal OA in laparoscopic surgery for colon cancer.

## Introduction

Colorectal cancer is one of the most common diseases and has a high incidence and mortality rate worldwide [1]. More than 30 years have passed since the first laparoscopic colectomy was reported [2], and it is now widespread worldwide. Laparoscopic colectomy includes two type different anastomoses procedures: intracorporeal and extracorporeal. The first widely used procedure was the extracorporeal anastomosis, which is performed after specimen extraction from a mini-laparotomy. The reason for its popularity is that it could be adapted from the anastomotic technique of open surgery [3] and is more easily accepted by surgeons. Recently, intracorporeal anastomosis is being actively introduced with the evolution of laparoscopic linear stapler devices and the improved skills of laparoscopic surgeons. The advantages of this technique include minimal dissection of the mesentery of the colon, minimal length of specimen extraction wound, and the risk of mesenteric twisting is low [3, 4]. A total of two large randomized clinical trials (RCTs) [5, 6] reported that intracorporeal anastomosis has shorter length wounds, quicker recovery of bowel function, and less postoperative analgesia than extracorporeal anastomosis. In previous reports, overlap anastomosis (OA) has been performed as the most standard method of intracorporeal anastomosis. To avoid narrowing the anastomotic area, the closure of the enterotomy created for insertion of the stapler is often performed with sewn running sutures of the monofilament [5–7]. The double-layer handsewn closure is often used because of significantly lower incidence of anastomotic leakage and shorter postoperative hospital stay than the single layer closure [7]. The issue is that it takes time to master the complicated suturing technique. However, the stapled closure of enterotomy is a simple, quick, and uniform closure of enterotomy with high quality. To achieve this, it is necessary to ensure that the anastomotic area is not narrowed compared with that of handsewn closure of enterotomy.

Maximum intensity projection is a postprocessing technique for computed tomography images that selects pixels with the highest signal density from specific cross-sectional data and projects them as a two-dimensional image (maximum intensity projection-computed tomography, MIP-CT) [8]. It specifies an arbitrary viewpoint in the 3D volume data and displays the maximum intensity values of voxels (3D pixels) viewed from that direction on the projection plane. This highlights structures with high density. This can be used to measure the anastomotic area.

The purpose of this study is to create two porcine in vitro colon models of stapled versus handsewn closure of enterotomy in intracorporeal OA and to compare the anastomotic area using MIP-CT, anastomotic time, and anastomotic strength.

## Materials and methods

### Study design

**Fig. 1:**
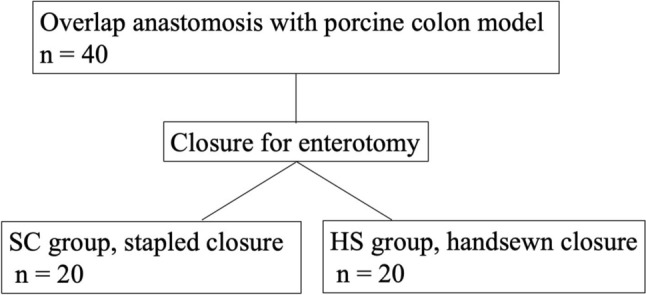
Flow chart illustrating the overlap anastomosis with porcine colon model are shown

A total of 40 porcine in vitro colon OA models was used to generate (20 in the stapled closure, SC group and 20 in the handsewn closure with monofilament, HC group, in which the enterotomy was closed) (Fig. 1). Porcine colons were obtained from a slaughterhouse (Tokyo Shibaura Zouki, Tokyo, Japan). Samples were immediately harvested from slaughtered animals and transported to the laboratory within 24 h. Colons were cut into 20-cm sections for the anastomosis experiment using a linear stapler (Endo GIA™, 60-mm triple row cartridge). This study was approved by the institutional animal ethics committee of our institute (approval no. T19-0054).

### Overlap anastomosis procedure and anastomotic time

**Fig. 2:**
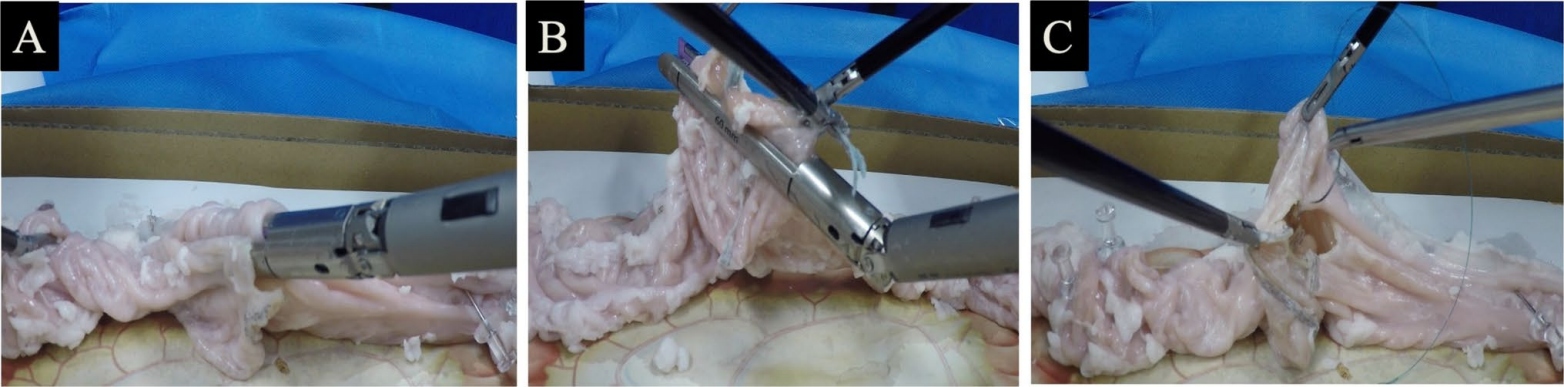
**A** The porcine in vitro colon is placed 6 cm longitudinally across the colon, an enterotomy is created, a stapler is inserted, and side-to-side anastomosis is performed. **B** The stapled closure group elevates the enterotomy and closes it with a single stapler. **C** The handsewn closure group closes it with a double layer using monofilament

All procedures were performed in a laparoscopic dry box environment. A porcine in vitro colon is placed 6 cm longitudinally across the colon; a small incision was made as an entry hole for inserting a linear staple near the staple line of bowel. Similarly, an entry hole was created at 6 cm from the staple line of bowel. The 60-mm stapler jaw was inserted into both entry holes in coordination with the assistant surgeon. Overlap anastomosis was performed using a linear stapler (Fig. 2A). In the SC group, the enterotomy is elevated and closed with a single stapler (Fig. 2B). In the HC group, it is closed with a double layer by laparoscopic running sewing that used a 3-0 polydioxanone (PDS)* II (Fig. 2C). The time from the creation of the enterotomy to the closure of the enterotomy was defined as the anastomotic time.

### Measurement of anastomotic area

**Fig. 3:**
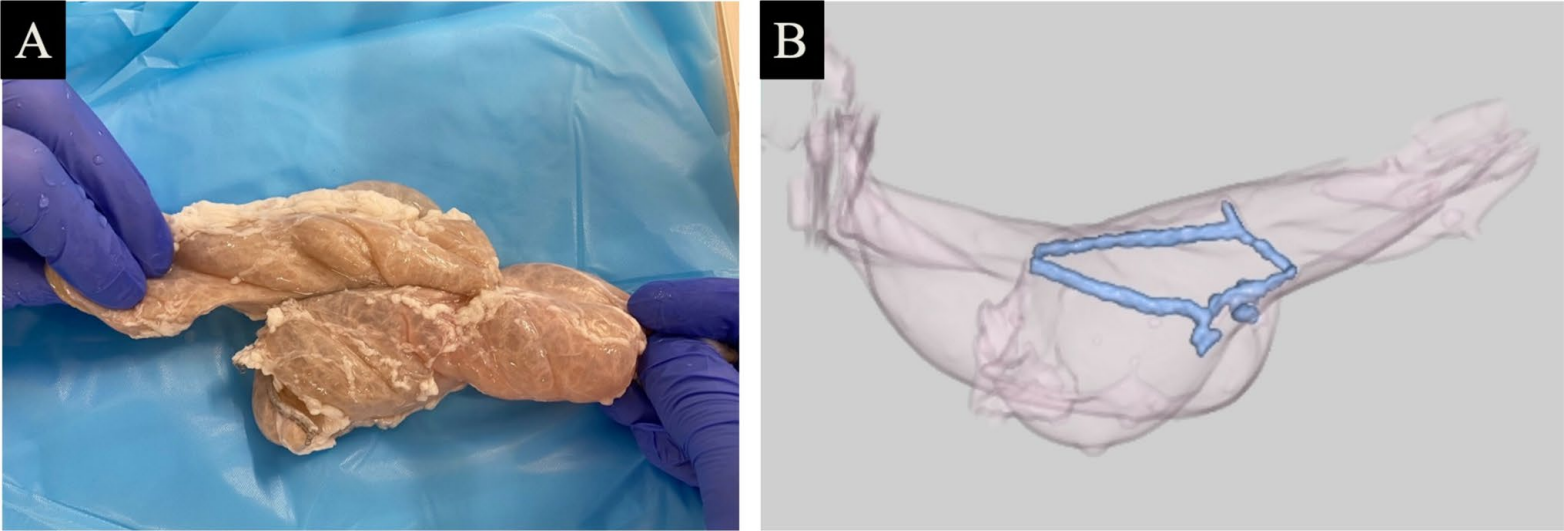
**A** The completed overlap anastomosis is filled with 20 ml of saline solution mounted on a fixed table and imaged using maximum intensity projection-computed tomography (MIP-CT). **B** The anastomotic area was measured by plotting and tracing a temporary line between the cut edges of the staple line in 1-mm increments with MIP-CT

The completed overlap anastomosis was filled with 20 ml of saline solution (Fig. 3A), mounted on a fixed table, and imaged using 128-row multidetector computed tomography (GE Healthcare, Little Chalfont, UK) at 1-mm slice thickness with the region of interest set to the anastomosis. The data were analyzed using a workstation three-dimensional image analysis system volume analyzer (SYNAPSE VINCENT; Fujifilm Corporation, Tokyo, Japan). The MIP technique is well suited for clearly visualizing the contour of the intestinal anastomosis, and settings were adjusted to maximize contrast with the lumen and surrounding structures, especially at the anastomosis (Fig. 3B). Using software to automatically recognize staples has a limited ability for extraction; therefore, the engineer reviewed the horizontal section database further and reconstructed a detailed staple image by plotting and tracing the cut end of the staple line in HC group with 1-mm increments. Due to software limitations in extraction, the technician further examined the horizontal cross-section database and reconstructed a detailed staple image by plotting and tracing the cut end of staple line in HC group with 1-mm increments; the anastomotic area was measured by plotting and tracing a temporary line between the cut edges of the staple line in 1-mm increments. To improve measurement accuracy, measurements were performed by two independent examiners.

### Leakage pressure test

The connecting tube connected to an infusion pump and a pressure recorder (Handy manometer PG-100, Copal Electronics, Tokyo, Japan) via a pressure transducer was placed in the bowel of the completed OA 5 and ligated with a thread. The OA was immersed in water in a tank and air was injected into the OA at a rate of 30 ml/min. Intraluminal pressure was continuously recorded. Leakage pressure was defined as the pressure at which air leakage from the anastomosis was first observed.

### Statistical analysis

All statistical analyses were performed using SPSS version 29 (IBM, Armonk, NY, USA). The Mann–Whitney *U* and chi-squared tests were used to compare continuous variables between the two groups. Results were presented as means and standard deviations. A *p*-value < 0.05 in the univariate analysis was considered statistically significant.

## Results

**Fig. 4:**
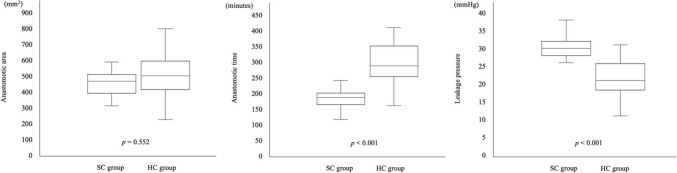
The anastomotic area showed no significant difference between the SC group and HC group. The anastomotic time was significantly shorter in the SC group than in the HC group. The leakage with was significantly higher in the SC group than in the HC group

The anastomotic area showed no significant difference between the SC group and HC group (474.0 ± 105.0 mm^2^ versus 502.6 ± 155.6 mm^2^, *p* = 0.552). The anastomotic time was significantly shorter in the SC group than in the HC group (185.9 ± 38.3 s versus 292.4 ± 67.8 s, *p* < 0.001). The leakage pressure was significantly higher in the SC group than in the HC group (30.1 ± 3.8 mmHg versus 21.6 ± 5.3 mmHg, *p* < 0.001) (Fig. 4).

## Discussion

The purpose of this study was to compare stapled versus handsewn closure of enterotomy in OA, the most frequently used intracorporeal anastomosis in laparoscopic colectomy. The findings of this study using porcine in vitro colon model denote that SC in OA has a similar anastomotic area, significantly shorter anastomotic time and significantly higher leakage pressure compared with HC. To the best of our knowledge, this is the first study that stapled versus handsewn closure of enterotomy in OA.

Bowel reconstruction is one of the most important steps in the surgery for colon cancer. In recent years, various types of intracorporeal anastomosis have been introduced in minimally invasive surgery for colon cancer [9]. In previous reports comparing intracorporeal anastomosis with extracorporeal anastomosis, it was initially reported that an antiperistaltic anastomosis, which is called functional end- to-end anastomosis (FEEA), is the most common type of anastomosis. The advantage of FEEA is that surgeons are accustomed to using it in open surgery, and its disadvantage is that it requires extensive bowel manipulation. By contrast, Delta anastomosis [10–12], which is based on gastric surgery techniques, has recently been used in colon surgery. This technique has the advantage of short anastomotic time; however, it has the potential for mesial adipose tissue to intervene in the anastomosis because the enterotomy is created on the mesenteric side. However, it has problems such as the possibility of mesial adipose tissue intervening in the anastomosis, a high risk of penetrating the bowel wall with the stapler tip while making the anastomosis, and a narrowing of the anastomotic area [11, 12]. By contrast, OA results in a lower risk of pinching of the mesial adipose tissue because the complete contramesenteric side is anastomosed. The thickness of the stapled tissue in the anastomosis results in less fatty tissues obstructing the field of view. It also has the advantage of precise recognition of the bowel wall during enterotomy closure. Under conditions of intracorporeal manipulation, the features of isoperistaltic methods, including easier alignment of the anastomotic axis with the bowel axis, likely have more advantages than those of FEEA. These features may contribute to the lower incidence of anastomotic leakage in OA [10, 13]. For these reasons, OA has also been used in recent large RCTs [5, 6]. HC is often used to avoid narrowing of the anastomotic area. In addition, reports that double-layer closure has a significantly lower incidence of anastomotic leakage and a shorter postoperative period in enterotomy closure methods [7] further complicate the surgical process. However, the laparoscopic suturing technique is technically more difficult and complicated. Therefore, in our study, the standard deviation of anastomotic area is larger in HC, which is susceptible to differences in individual surgeon competence, than in SC. Conversely, in SC, it was possible to obtain an anastomotic area comparable to that of HC in a stable manner by using a linear stapler. If there is no difference in anastomotic area, SC without suturing technique should be simpler and easier than HC with suturing technique. Compared with HC, SC with a surgical device has a smaller standard deviation of anastomotic area, and a more uniform and stable anastomotic area can be obtained.

The most technically difficult procedure for laparoscopic surgeons is the suturing technique, which requires time to become proficient. The learning curve for OA with handsewn closure of enterotomy was reported to require 20 cases [14]. This is one of the reasons why the duration of operation time for intracorporal anastomosis is longer than that for extracorporeal anastomosis [5]. The results of this study showed that the anastomotic time was significantly shorter in SC compared with HC, and the use of a stapler can simplify the procedure and eliminate differences in surgeon skill. A simple procedure that does not depend on the surgeon’s skill is a prerequisite for a good surgical procedure. The long time required to close the enterotomies also increases the frequency of exposure to intraperitoneal spillage of bowel content, leading to increased incidence of increased organ/space surgical site infections [15]. From this perspective, SC is superior to HC. However, it should be noted that our study was conducted in an in vitro colon model. In actual clinical practice, it is necessary to consider the influence of the patient’s body mass index and the amount of mesenteric adipose tissues. In addition, the skill of the surgical assistant during laparoscopic surgery can affect the anastomosis.

In previous RCTs, anastomotic leakage occurs with a certain frequency in intracorporeal anastomosis (4.0%) [5]. Other reports indicate that intracorporeal anastomosis (8.6%) tends to have more anastomotic leakage than extracorporeal anastomosis (2.9%) [6]. A previous report also reported a lower morbidity rate in anastomoses with a triple row of staples in terms of anastomotic leakage [16], and this study also used a triple row of staples. The results of this study showed that SC had significantly higher leakage pressure than HC. Anastomotic quality is multifactorial—influenced by physiological parameters such as tension between the two connected portions of the gastrointestinal tract, vascular perfusion, peristalsis, inflammatory responses, and the mechanical strength of the formed tissues [17, 18]. Although leakage pressure is not everything, the leakage test results obtained in this study are considered important results suggesting the superiority of SC compared with HC.

This in vitro study has several limitations. First, the number of subjects was small. Second, the MIP technique using 128-row multidetector computed tomography for quantification of anastomotic area is not previously reported. It is important to validate this new attempt. Further studies are needed to evaluate the results. Third, it is not a study using a colon of human specimens and is an in vitro study in an environment different from that of actual clinical practice. In the future, large-scale comparative studies between SC and HC in actual clinical settings will be necessary.

## Conclusions

The findings of this study using porcine in vitro colon model showed that in OA, the anastomotic area was similar, anastomotic time was significantly shorter, and leakage pressure was significantly higher in SC compared with HC. The results suggest that SC may be superior to HC when performing intracorporeal OA in laparoscopic surgery for colon cancer.

## Data Availability

No datasets were generated or analyzed during the current study.
